# ‘Breathing Fire’: Impact of Prolonged Bushfire Smoke Exposure in People with Severe Asthma

**DOI:** 10.3390/ijerph19127419

**Published:** 2022-06-16

**Authors:** Tesfalidet Beyene, Erin S. Harvey, Joseph Van Buskirk, Vanessa M. McDonald, Megan E. Jensen, Jay C. Horvat, Geoffrey G. Morgan, Graeme R. Zosky, Edward Jegasothy, Ivan Hanigan, Vanessa E. Murphy, Elizabeth G. Holliday, Anne E. Vertigan, Matthew Peters, Claude S. Farah, Christine R. Jenkins, Constance H. Katelaris, John Harrington, David Langton, Philip Bardin, Gregory P. Katsoulotos, John W. Upham, Jimmy Chien, Jeffrey J. Bowden, Janet Rimmer, Rose Bell, Peter G. Gibson

**Affiliations:** 1 School of Medicine and Public Health, The University of Newcastle, Callaghan, NSW 2308, Australia; tesfalidet.beyene@newcastle.edu.au (T.B.); erin.harvey@newcastle.edu.au (E.S.H.); megan.jensen@newcastle.edu.au (M.E.J.); vanessa.murphy@newcastle.edu.au (V.E.M.); liz.holliday@newcastle.edu.au (E.G.H.); anne.vertigan@health.nsw.gov.au (A.E.V.); 2 Department of Respiratory and Sleep Medicine, John Hunter Hospital, Newcastle, NSW 2305, Australia; vanessa.mcdonald@newcastle.edu.au (V.M.M.); john.harrington@health.nsw.gov.au (J.H.); 3 Sydney School of Public Health and University Centre for Rural Health, Faculty of Medicine and Health, University of Sydney, Sydney, NSW 2006, Australia; joseph.vanbuskirk@sydney.edu.au (J.V.B.); geoffrey.morgan@sydney.edu.au (G.G.M.); edward.jegasothy@sydney.edu.au (E.J.); ivan.hanigan@sydney.edu.au (I.H.); 4 School of Nursing and Midwifery, The University of Newcastle, Callaghan, NSW 2308, Australia; 5 School of Biomedical Sciences and Pharmacy, The University of Newcastle, Callaghan, NSW 2308, Australia; jay.horvat@newcastle.edu.au; 6 Tasmanian School of Medicine, Menzies Institute for Medical Research, University of Tasmania, Hobart, TAS 7000, Australia; graeme.zosky@utas.edu.au; 7 Department of Speech Pathology, John Hunter Hospital, Newcastle, NSW 2305, Australia; 8 Department of Thoracic Medicine, Concord Hospital, Concord, NSW 2139, Australia; matthew.peters@health.nsw.gov.au (M.P.); christine.jenkins@sydney.edu.au (C.R.J.); 9 Concord Clinical School, University of Sydney, Concord, NSW 2006, Australia; claude.farah@sydney.edu.au; 10 School of Medicine, Western Sydney University, Campbelltown Hospital, Campbelltown, NSW 2560, Australia; connie.katelaris@health.nsw.gov.au; 11 Faculty of Medicine, Nursing and Health Sciences, Monash University, Clayton, VIC 3800, Australia; davidlangton@phcn.vic.gov.au; 12 Department of Thoracic Medicine, Frankston Hospital, Frankston, VIC 3199, Australia; 13 Lung and Sleep Medicine, Monash University and Medical Centre, Clayton, VIC 3168, Australia; philip.bardin@monash.edu; 14 St George Specialist Centre, Kogarah, NSW 2217, Australia; drgpk@stgeorgesc.com.au; 15 St George and Sutherland Clinical School, University of New South Wales, Sydney, NSW 2052, Australia; 16 Woolcock Institute of Medical Research, Glebe, NSW 2037, Australia; janet.rimmer@svha.org.au; 17 Department of Respiratory Medicine, Princess Alexandra Hospital, Woolloongabba, QLD 4102, Australia; j.upham@uq.edu.au; 18 Diamantina Institute, The University of Queensland, Woolloongabba, QLD 4102, Australia; 19 Department of Respiratory and Sleep Medicine, Westmead Hospital, Westmead, NSW 2145, Australia; jimmy.chien@sydney.edu.au; 20 School of Medicine, The University of Sydney, Sydney, NSW 2050, Australia; 21 Respiratory and Sleep Services, Flinders Medical Centre, Flinders University, Bedford Park, SA 5042, Australia; jeff.bowden@sa.gov.au; 22 St Vincent’s Clinic, Darlinghurst, NSW 2010, Australia; 23 Asthma Australia, Melbourne, VIC 3003, Australia; rbell@asthma.org.au

**Keywords:** severe asthma, particulate matter, wildfire smoke, bushfire smoke

## Abstract

Wildfires are increasing and cause health effects. The immediate and ongoing health impacts of prolonged wildfire smoke exposure in severe asthma are unknown. This longitudinal study examined the experiences and health impacts of prolonged wildfire (bushfire) smoke exposure in adults with severe asthma during the 2019/2020 Australian bushfire period. Participants from Eastern/Southern Australia who had previously enrolled in an asthma registry completed a questionnaire survey regarding symptoms, asthma attacks, quality of life and smoke exposure mitigation during the bushfires and in the months following exposure. Daily individualized exposure to bushfire particulate matter (PM_2.5_) was estimated by geolocation and validated modelling. Respondents (*n* = 240) had a median age of 63 years, 60% were female and 92% had severe asthma. They experienced prolonged intense PM_2.5_ exposure (mean PM_2.5_ 32.5 μg/m^3^ on 55 bushfire days). Most (83%) of the participants experienced symptoms during the bushfire period, including: breathlessness (57%); wheeze/whistling chest (53%); and cough (50%). A total of 44% required oral corticosteroid treatment for an asthma attack and 65% reported reduced capacity to participate in usual activities. About half of the participants received information/advice regarding asthma management (45%) and smoke exposure minimization strategies (52%). Most of the participants stayed indoors (88%) and kept the windows/doors shut when inside (93%), but this did not clearly mitigate the symptoms. Following the bushfire period, 65% of the participants reported persistent asthma symptoms. Monoclonal antibody use for asthma was associated with a reduced risk of persistent symptoms. Intense and prolonged PM_2.5_ exposure during the 2019/2020 bushfires was associated with acute and persistent symptoms among people with severe asthma. There are opportunities to improve the exposure mitigation strategies and communicate these to people with severe asthma.

## 1. Introduction

Wildfires are an increasing global problem, where they are burning for a longer period, with more intensity and more frequently [1]. Wildfires cause high levels of air pollution through the generation of smoke and dust particles [2], and are associated with extensive health impacts [3]. Wildfire smoke, produced through biomass burning, includes pollutants, such as particulate matter (PM), carbon monoxide, nitrogen oxides and volatile organic compounds [4]. Suspended fine PM, which is less than or equal to 2.5 µm in diameter (PM_2.5_), is prominent and the most important in terms of health. PM_2.5_ can be absorbed deeply in the respiratory tract, causing inflammation, and can enter the blood stream to produce a range of serious health effects [5,6].

The impact of short-term wildfire smoke exposure on asthma, respiratory and cardiovascular diseases is reported. Short-term wildfire smoke exposure is associated with increased hospital attendance for asthma attacks [7,8,9,10]. A systematic review of Australian studies [7] identified significant associations between wildfire smoke and respiratory impacts, with the effects greatest on the day of exposure. For asthma, the seven studies included identified significant associations between wildfire smoke PM or smoke event days and emergency department presentations or hospital admissions. Wildfire-specific PM_2.5_ appears to have a greater effect, compared to PM_2.5_ from other sources [10,11,12]. The enhanced effect may be due to a range of factors, including greater exposure peaks, more intense cumulative exposure and differences in the chemical composition of the PM_2.5_ [13,14].

During the summer of 2019/2020, Australia experienced intense and prolonged wildfires (bushfires) that were unprecedented in the duration of burning. Over 10 million people across three states were exposed to bushfire smoke for a period of several months [7,15]. The burnt area was extensive [16]. In the Eastern states, the highest estimated population-weighted daily average exposure level to PM_2.5_ was 98.5 μg/m^3^ on 14 January 2020. This exceeded the national air quality 24-h standard (25 μg/m^3^) and was greater than fourteen times the historical population-weighted mean 24-h PM_2.5_ value (6.8 μg/m^3^) [15]. Smoke-related health costs were calculated at AU$1.95 billion, more than nine times the median of the nineteen previous seasons [17]. Statistical modelling from the 2019/2020 wildfire period estimated that the acute effects of the smoke were responsible for 417 (95% CI, 153–680) excess deaths, 1124 (95% CI, 211–2047) hospitalizations for cardiovascular problems, 2027 (95% CI, 0–4252) hospitalizations for respiratory problems and 1305 (95% CI, 705–1908) presentations to emergency departments for asthma [15]. Adverse health effects were reported by people with and without pre-existing respiratory conditions, with the effects more commonly reported in those with respiratory conditions [18,19].

The impacts of prolonged wildfire smoke exposure, and in people with severe asthma, have not been described. Studies in this area are required to inform public health messaging for future wildfire events and ensure the appropriate management of individuals with severe asthma, both outside of and during wildfire events. This study investigated the immediate and ongoing health impacts of prolonged wildfire (bushfire) smoke exposure on adults with severe asthma. We also characterized the information sources and participants’ actions to mitigate the effects of bushfire smoke exposure. This study specifically addressed the following questions: 1. Were individual participant asthma outcomes different between the 2019/2020 bushfire exposure period and the preceding less severe 2018/2019 bushfire exposure period? 2. Were there differences in the patterns of acute and persisting symptoms experienced by the participants due to the 2019/2020 bushfire period? 3. Did individual participant characteristics prior to the 2019/2020 bushfire exposure period predict persisting symptoms following the exposure period?

## 2. Materials and Methods

### 2.1. Study Design

We used the established severe asthma registry cohorts to identify the impact of the 2019/2020 bushfires on the participants with severe asthma. We compared the data on asthma attacks and health care utilization for the 2019/2020 bushfire period, and the same period in 2018/2019. The bushfire-related symptoms, actions taken to mitigate exposure and persistent asthma symptoms after the 2019/2020 bushfire were assessed by means of a questionnaire. The daily individualized exposure to the bushfire PM_2.5_ was estimated by geolocation and validated modelling and related to asthma outcomes.

The Australasian Severe Asthma Registry (ASAR) enrolls adults with severe refractory asthma and a non-severe asthma comparison group [20], and the Australian Mepolizumab Registry (AMR) enrolls adults and adolescents with severe uncontrolled eosinophilic asthma treated with mepolizumab [21]. The participants were carefully characterized at their enrolment visit, which was prior to the wildfire period, and reassessed each 6 months. The participants (*n =* 305), who were enrolled in the registries at centers (*n =* 10) in Brisbane (Queensland), Newcastle and Sydney (New South Wales), Melbourne (Victoria) and Adelaide (South Australia) were contacted by telephone and invited to participate.

The criteria for enrolment in each of the registries include a doctor’s diagnosis of asthma with confirmed objective evidence of variable airflow obstruction, and the European Respiratory Society/American Thoracic Society taskforce definition for severe asthma; remaining poorly controlled despite maximal asthma therapy, or who lose control upon the tapering of this treatment [22]. Participants had optimized management skills and assessment and management of comorbidities and triggers.

### 2.2. Data Collection: Clinical Data and Health Outcomes

The asthma registry data included demographics, asthma and medication characteristics and comorbidities. Asthma attacks, oral corticosteroid (OCS) use and health care utilization collected in the preceding bushfire period (2018/2019) were extracted.

Questionnaire survey: Data related to the 2019/2020 Australian bushfire period were collected via a self-report survey of the participants after the bushfire period had ended (survey commenced 19 March 2020 and closed 31 May 2020). The survey was completed online, by telephone or on paper, and used the REDCap electronic data capture tools hosted at the Hunter Medical Research Institute, Newcastle, Australia [23].

The survey (available from https://www.severeasthma.org.au/wp-content/uploads/2021/07/Bushfire-severe-asthma-survey-v2-10032020_CRE-UPLOAD.pdf) included items from prior surveys [19,24], and questions related to symptoms, exposure advice and mitigation strategies, and the place of residence during the bushfire period. The participants provided their primary residential location (street address) during the bushfire period, and the addresses and dates for up to three additional locations where they had stayed during the bushfire period. The final component of the survey comprised an assessment of ongoing symptoms in the months following the bushfire smoke exposure period. The participants were asked about the symptoms they were experiencing (respiratory and non-respiratory) during the 4 weeks prior to the survey completion, and their level of asthma control.

### 2.3. Data Collection: Exposure Measures

The exposure period was defined as the 2019/2020 bushfire period (1 October 2019 to 29 February 2020; 152 days). The PM_2.5_ exposure during this period was calculated for the participants located within the Melbourne Region, Victoria, and the Sydney Greater Metropolitan Region (GMR) study region, New South Wales, which includes the metropolitan areas of Sydney, Newcastle and Wollongong (Supplementary Materials, Figures S1 and S2).

We obtained the measured daily 24-h mean PM_2.5_ data from the fixed-site government air quality monitoring stations within the study regions (NSW Department of Planning, Industry and Environment and Environmental Protection Agency Victoria) [25,26]. The measured daily data were interpolated within the study regions using an inverse distance weighting procedure to estimate the daily PM_2.5_ (µg/m^3^) exposure concentration for the participant’s residential location [27].

We identified the bushfire smoke days from a database produced by Van Buskirk, J., & Hanigan, I., 2021, Bushfire specific PM_2.5_ surface at participant’s residential locations for 2006–2020, downloaded from https://cloudstor.aarnet.edu.au/plus/f/5638670382 (accessed on 10 December 2020), based on government data and satellite imagery. The bushfire days were defined following a validation protocol designed for Australian bushfires [28] as days when: the entire study region’s 24-h average of PM_2.5_ concentration exceeded the 95th percentile (based on the period 28 January 2014 to 31 December 2018 for the Melbourne region and 1 January 2000 to 31 December 2018 for the Sydney GMR), and there was visual confirmation of fire for that day or up to three days before or after via satellite imagery. The elevated PM_2.5_ levels on these days could be attributed to bushfire smoke [27]. To control for spatial variability in the region, an additional requirement was that the interpolated PM_2.5_ reading for each participant’s residential address also exceeded the 95th percentile for the region (Supplementary Materials).

For each participant, their daily PM_2.5_ concentration levels over the 152-day study period were averaged to obtain their mean PM_2.5_ (µg/m^3^) exposure. The participant’s peak PM_2.5_ (µg/m^3^) was determined as the maximum 24-h concentration value to which a participant was exposed during the 152-day study period. The median PM_2.5_ values were used to categorize the participants according to their levels of exposure: mean daily (≤16 and >16 µg/m^3^) and peak PM_2.5_ (≤115 and >115 µg/m^3^). The exposure was further categorized according to the total bushfire days (≤41 and >41 days) and the maximum consecutive bushfire days (≤10 and >10 days), based on data distribution.

### 2.4. Statistical Analysis

Key comparisons were: 1. asthma outcomes during the 2019/2020 and 2018/2019 bushfire periods; 2. symptoms during and after the 2019/2020 bushfire period (Supplementary Materials). Bivariate analyses were performed to assess the crude (unadjusted) relationship between the bushfire smoke exposures and self-reported symptoms. Multivariable analyses were performed to estimate the total, unconfounded effect of bushfire-related smoke exposure on self-reported symptoms. Potential confounders were identified using reported evidence and visualized by constructing a directed acyclic graph (DAG) [29], which specified the assumed causal relationships between exposure, outcome and covariates [30] (Supplementary Materials, Figure S3). Using the DAG, we identified the minimum adjustment set required to estimate the unconfounded effect of bushfire smoke exposure on self-reported symptoms. The minimum adjustment set included one potential confounding variable: whether actions were taken during the bushfire period (stayed indoors/avoided going outdoors, kept windows and doors shut when inside, used a facemask, used an indoor air cleaner/purifier in your home, avoided exercising outdoors and relocated to another area). Poisson regression models with robust standard errors were used to estimate the effect of the bushfire smoke events or bushfire-related PM_2.5_ on self-reported symptoms during and following the bushfire period. Estimates were expressed as crude relative risk (cRR) and adjusted relative risks (aRR) with 95% confidence intervals (CI). All adjusted models included the confounding variable identified as the minimum adjustment set. We also conducted stratified analyses to assess the relationship between bushfire-related smoke exposure and symptoms within categories of putative effect modifiers: sex, monoclonal antibody use at the pre-bushfire visit and asthma symptom control at the pre-bushfire visit. The asthma symptom control was categorized using the ACQ-5 score (controlled asthma: ACQ-5 < 1.5 vs. uncontrolled asthma: ACQ-5 ≥ 1.5). Effects estimated within the strata were reported as aRR with 95% CI, together with the type III *p*-value for interaction between the exposure and relevant effect modifier, estimated using a separate model. The statistical analysis was performed using STATA version 16 (TX, USA). A *p*-value of <0.05 was considered statistically significant. The PM_2.5_ line graphs were prepared using the R package “ggplot2”.

## 3. Results

**Figure 1:**
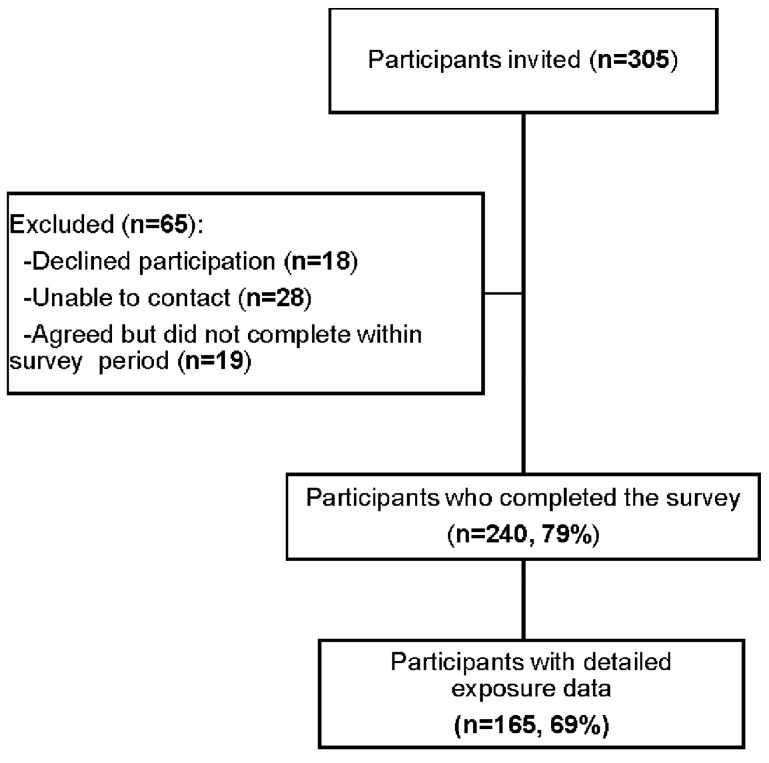
Participant flow diagram.

Of 305 registry participants, 240 (79%) completed the survey (Figure 1). Detailed bushfire smoke exposure data were available for 165 (69%) participants. The survey completion was a median (Q1, Q3) 48 (34, 66) days after the 2019/2020 bushfire period.

### 3.1. Bushfire Smoke Exposure

**Figure 2:**
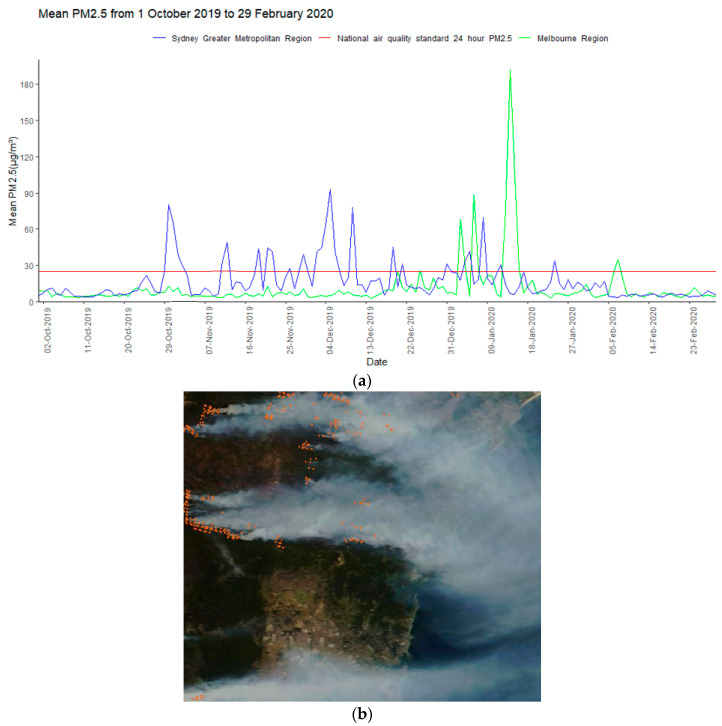
Bushfire smoke exposure during the 2019/2020 bushfire period. Participant exposure was assessed using PM_2.5_ measures from fixed site monitors and geolocated for participant address (Panel (**a**)). Confirmation of bushfire activity was obtained from images as seen by the Moderate Resolution Imaging Spectroradiometer Terra satellite (exemplar image shown in Panel (**b**)). Bushfire exposure days were identified using a method validated for Australian settings based on data from panels (**a**,**b**) using high level PM_2.5_ exposure together with satellite image confirmation of bushfire activity. (**a**) Population-weighted mean daily PM_2.5_ concentration in the Sydney Greater Metropolitan Region (New South Wales) and Melbourne Region (Victoria) during the 2019/2020 bushfire period (1 October 2019 to 29 February 2020); (**b**) Fire hot spots and smoke plumes in the Sydney region, as seen by the Moderate Resolution Imaging Spectroradiometer Terra satellite on 4 December 2019. The orange spots indicate fires.

Participants with asthma experienced a median daily average PM_2.5_ exposure over the bushfire period of 16.4 (11.3, 16.7) μg/m^3^ and median peak PM_2.5_ exposure of 115.0 (101.3, 191.7) μg/m^3^. There were 55 and 6 bushfire exposure days for the Sydney GMR and Melbourne region, respectively, during the 152-day exposure period. The mean PM_2.5_ was 32.5 μg/m^3^ on bushfire days compared with 9.9 μg/m^3^ on non-bushfire days. The daily average PM_2.5_ concentrations were above the national air quality 24-h standard (25 μg/m^3^) on 23 of the 152 days (Figure 2).

### 3.2. Demographic and Clinical Characteristics

There were 222 respondents with severe asthma and 18 with non-severe asthma. The participants had a median age of 63.5 (53.8, 71.3) years and 145/240 (60.0%) were female (Table 1). Nearly one third (76/240, 31.7%) were in paid employment and 108/240 (45.0%) were retired. Most (62.7%) of the participants were never-smokers.

The participants had a median asthma duration of 34.9 (18.8, 52.5) years. Prior to the 2019/2020 bushfire period, 46/225 (20.4%) of the participants had specifically identified smoke/wood fire/bushfires as asthma triggers. At the pre-bushfire asthma registry assessment visit, participants had a median Asthma Control Questionnaire-5 (ACQ-5) score of 1.4 (0.6, 2.2), mean (SD) post-bronchodilator FEV_1_ percent of predicted 69.4 (21.5) and mean FEV_1_/FVC ratio of 0.6 (0.1). Approximately two thirds of the participants were receiving monoclonal antibody treatment (mepolizumab, omalizumab or benralizumab) for severe asthma, and 21% were using maintenance OCS. Comorbidities were common and included: allergic rhinitis (53.0%); gastro-esophageal reflux disease (46.6%); cardiovascular disease (35.5%); psychiatric conditions (anxiety/depression/other) (34.6%); eczema (24.4%) and nasal polyps (23.1%).

### 3.3. Symptoms during Bushfire Smoke Exposure

**Figure 3:**
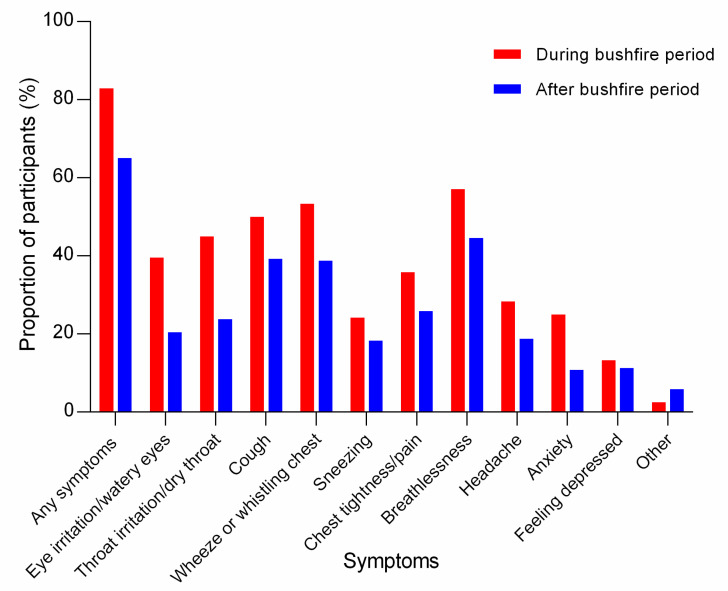
Symptoms reported by participants during and following the 2019/2020 bushfire period.

Most (199/240, 82.9%) of the participants experienced symptoms during the 2019/2020 bushfire period. Breathlessness (137/240, 57.1%), wheeze or whistling chest (128/240, 53.3%), cough (120/240, 50.0%) and throat irritation/dry throat (108/240, 45.0%) were most prevalent (Figure 3). Three quarters (146/199, 73.4%) of the participants attributed their symptoms to the bushfire smoke exposure. Less than half (87/199, 43.7%) sought advice from a health professional for the symptoms; with general practitioners being the most commonly consulted professional (77/199, 38.7%). Approximately one third of the participants (68/199, 34.2%) took time off work or daily activities due to symptoms. Prolonged exposure to bushfire smoke reduced their capacity to participate in usual activities (155/240, 64.6%), resulted in cancelling an important sporting or social engagement (87/240, 36.3%) or resulted in being sick for greater than one week (86/240, 35.8%). Intensity of exposure was not associated with symptoms during the bushfire period (Table 2).

### 3.4. Comparison of Asthma between the 2019/2020 Bushfire Period and the 2018/2019 Bushfire Period

Participants’ exposure to bushfire smoke PM_2.5_ was significantly greater during the 2019/2020 bushfire period compared to the 2018/2019 period (Table 3). More of the participants required an unscheduled doctor visit for an asthma attack during the 2019/2020 bushfire period than during the 2018/2019 bushfire period (*p* < 0.05).

### 3.5. Comparison of Asthma before and during the 2019/2020 Bushfire Period

During the 2019/2020 bushfire period, most of the participants required increased reliever use (176/240, 73.3%) and 106/240 (44.2%) started or increased OCS for an asthma attack. The proportion of participants who required an unscheduled doctor visit for an asthma attack during the bushfire period was increased at 30.8%, compared to 11.5% at the pre-bushfire visit (*p* < 0.001), with the number of doctor visits also increased. No statistically significant difference was seen in the other healthcare utilization parameters. The proportion of participants who required hospital admission for an asthma attack during the bushfire period was too low to permit analysis.

### 3.6. Persistent Symptoms Following the 2019/2020 Bushfire Period

Approximately two-thirds (156/240, 65.0%) of the participants reported persistent symptoms following the 2019/2020 bushfire exposure period. Commonly reported persistent symptoms were: breathlessness (107/240, 44.6%); cough (94/240, 39.2%) and wheeze or whistling chest (93/240, 38.8%) (Figure 3).

Persistent symptoms were associated with: female sex; smoking status; uncontrolled asthma before bushfire smoke exposure; impaired health-related QOL before exposure and the number of asthma trigger factors (Table 4 and Table 5). Adjusted analyses identified no association between the intensity and duration of bushfire smoke exposure and persistent symptoms. The sensitivity analysis showed no association between the bushfire smoke exposure and self-reported persistent symptoms (Supplementary Materials, Table S1). Participants who used monoclonal antibody therapy and experienced peak PM_2.5_ (>115 µg/m^3^) had reduced risk of symptoms following the bushfire period (persistent symptoms) (aRR 0.77; 0.60–0.99; *p* = 0.046) (Table 5). There was no significant association between bushfire smoke exposure with relative risk for self-reported persistent symptoms in males or females (Supplementary Materials, Table S2).

### 3.7. Smoke Exposure Risk Mitigation

To avoid or minimize exposure to the bushfire smoke during the 2019/2020 bushfire period (Supplementary Materials, Table S3), participants stayed indoors/avoided going outdoors (212/240, 88.3%), kept windows and doors shut when inside (223/240, 92.9%), used home air conditioners (173/240, 72.1%) and avoided exercising outdoors (199/240, 82.9%). The use of face masks (49/240, 20.4%) and home indoor air cleaner/purifiers (27/240, 11.3%) were less common. Relocation to another area was reported by 11/240 (4.6%) of the participants.

Less than half (107/240, 44.6%) of the participants reported receiving advice/information on how to manage their asthma during the bushfire period. Of these, two-thirds (70/107, 65.4%) received advice from a general practitioner, 39/107 (36.5%) from the news/current affairs stories and 27/107 (25.2%) from a respiratory/asthma specialist. Approximately half (124/240, 51.7%) of the participants reported receiving advice/information on avoiding/minimizing exposure to bushfire smoke during the period.

## 4. Discussion

Adults with severe asthma experienced intense and prolonged exposure to bushfire-related air pollution and suffered substantial impacts on their health and quality of life during the 2019/2020 Australian bushfire period. The majority reported acute respiratory and non-respiratory symptoms during the bushfire period, and persistent symptoms were experienced by many. This was despite most having undertaken smoke mitigation strategies to reduce exposure. However, few of them used face masks or air purifiers and less than half received advice regarding the management of their asthma during the bushfire period. Characteristics including female sex, having uncontrolled asthma and impaired quality of life before the bushfire smoke exposure were associated with persistent symptoms. Monoclonal antibody use for severe asthma appeared to protect against the persistence of symptoms.

Bushfire-related PM is specifically associated with poor asthma-related outcomes, such as hospitalizations or emergency department visits [31,32,33]. In this study, people with severe asthma were highly symptomatic during the 2019/2020 bushfire period, and had a high degree of health care utilization, with increased general practitioner visits and OCS use. However, hospitalizations and emergency department visits were infrequent. This was an unexpected result, which may be explained by participants’ ability to effectively manage asthma attacks and avoid hospitalization. Prior to the bushfire exposure, the participants had already optimized asthma self-management skills as part of their enrolment in the severe asthma registry. This is reflected in the self-initiation of OCS for an asthma attack by up to 40% during the bushfire period, and may have reduced their health care utilization. Almost two thirds of the participants experienced ongoing symptoms several months after the exposure, which was associated with a significant quality of life impairment. The mechanism of persistent symptoms requires further investigation.

Most of the participants took action during the 2019/2020 bushfire period to mitigate the effects of smoke exposure, yet still experienced acute and persistent symptoms. While staying indoors and keeping the doors/windows shut may be effective in the short-term, the home will eventually equilibrate with the external environment [34] during periods of prolonged exposure. Appropriately fitted P2/N95 masks are effective in filtering PM_2.5_ [35]; however, in this cohort, face masks were not commonly used. The use of air cleaners/purifiers was also uncommon. Increasing accessibility to these aids may benefit people with severe asthma.

Considering the inherent vulnerability of people with asthma to bushfire smoke exposure, it is notable that close to half of the severe asthma participants did not receive advice/information regarding bushfire smoke exposure minimization or managing their asthma during the bushfires. Furthermore, the knowledge sources differed. It is important that accurate and consistent health messaging occurs. Health care professionals should ensure that people with severe asthma are aware of the risks of exposure, effective mitigation strategies and adequately prepared to manage their asthma during bushfires.

There are some limitations to this study. Availability of exposure estimates for the study participants were limited by the location of, and accessibility, to air quality monitoring station and bushfire day data. The PM_2.5_ estimates for each region (Sydney GMR and Melbourne) differed in the number and spatial distribution of the monitors, affecting the distribution of estimates by region; however, this had a minimal effect on the individual participant PM_2.5_ estimates. Therefore, analyses were performed using merged data from the Sydney GMR and Melbourne regions. The smaller sample size combined with observed low variation in the exposure and outcome variables may have contributed to our inability to detect an association between the intensity/duration of exposure and the frequency of persistent symptoms. Moreover, the inclusion of a non-exposed comparator group may have increased our ability to detect an association between exposure and outcomes. However, owing to the widespread nature and duration of the exposure, the potential for recruitment of such individuals was limited. The Australian population is predominantly located on the Eastern/South-Eastern coasts of Australia. During the 2019/2020 Australian bushfire period, this extensive region was affected by the bushfires (including South-Eastern Queensland, New South Wales, Victoria and South Australia). As such, most of the population were exposed to bushfire smoke. The comparison of the individual participant outcomes during the 2019/2020 bushfire period and the preceding 2018/2019 period enabled us to compare outcomes in participants during both high and low exposure periods. The inclusion of a comparator group who are less symptomatic in general may have allowed us to observe an association between the exposure and symptoms. We attempted to recruit a non-severe asthma comparator group who were also representative of the registry. However, the response rate in this case prevented a statistical comparison.

## 5. Conclusions

This study investigated the immediate and ongoing impacts of the prolonged 2019/2020 bushfire smoke exposure period on adults with severe asthma. The participants’ information sources and exposure mitigation strategies were characterized. Despite most undertaking smoke exposure mitigation strategies, the majority of participants reported acute symptoms during the exposure period. Health care utilization for asthma attacks was increased during the 2019/2020 bushfire exposure period compared to the preceding less severe bushfire period. Many of the participants reported persistent symptoms following the exposure period. Characteristics including female sex, uncontrolled asthma and impaired quality of life before the exposure period were associated with persistent symptoms. Monoclonal antibody use appeared to be protective against the persistence of symptoms. In conclusion, the intense bushfire smoke exposure resulted in both acute and persistent symptoms among people with severe asthma. There are opportunities to improve strategies to mitigate exposure and communicate these to people with severe asthma. In particular, the impact of masks and air purifiers requires further evaluation. Future research is needed to assess the effectiveness of smoke-exposure risk mitigation strategies. Furthermore, longitudinal research is warranted to better understand the immediate and longer-term effects of bushfire smoke on people with severe asthma, and whether there are differences in outcomes compared to individuals with more mild asthma.

## Figures and Tables

**Table 1:** Demographic and clinical characteristics of study participants.

Variables	Number (%)
Respondents *n*	240
Age, years	63.47 (53.76, 71.30)
Sex (female)	145 (60.0)
**Smoking status**	
Never (%)/current (%)/ex-smoker (%)	62.7/2.1/35.2
Pack years (current/ex-smoker)	15.0 (5.3, 30.0)
Currently in paid employment	76 (31.7)
**ASTHMA CHARACTERISTICS**	
Severe asthma	222 (92.5)
Asthma duration, years	34.93 (18.76, 52.53)
Atopy, *n =* 191	143 (74.9)
*PRE-BUSHFIRE VISIT* *	
**Asthma control**	
ACQ-5 score	1.4 (0.6, 2.2)
**Post-bronchodilator lung function, *n =* 179**	
FEV_1_ % predicted	69.40 (21.52)
FVC % predicted	86.10 (16.50)
FEV_1_/FVC	0.62 (0.15)
**Exacerbations, *n =* 208**	
Requiring OCS, *n =* 208	83 (39.9)
Requiring hospital admission, *n =* 207	15 (7.2)
Requiring emergency department visit, *n =* 208	8 (3.8)
Requiring IV corticosteroids, *n =* 207	4 (1.9)
Requiring unscheduled Dr visit, *n =* 208	24 (11.5)
**Standardized Juniper Asthma Quality of Life Questionnaire, *n =* 198**	
AQLQ(S) overall score	5.45 (4.50, 6.34)
AQLQ(S) activity limitations	5.50 (4.36, 6.45)
AQLQ(S) symptoms	5.41 (4.50, 6.40)
AQLQ(S) emotional function	5.60 (4.40, 6.60)
AQLQ(S) environmental stimuli	5.75 (4.50, 6.50)
**Asthma treatments**	
Using maintenance OCS, *n =* 232	49 (21.12)
Using low dose macrolides, *n =* 231	27 (11.7)
Using monoclonal antibody, *n =* 234	156 (66.7)
Using ICS, *n =* 232	39 (16.81)
Using LABA, *n =* 232	3 (1.3)
Using LAMA, *n =* 232	101 (43.5)
Using ICS/LABA, *n =* 232	203 (87.5)
Using ICS/LABA/LAMA, *n =* 232	12 (5.1)
Using Theophylline, *n =* 232	11 (4.7)
Using Montelukast, *n =* 232	23 (9.9)

Data reported as *n*/*N* (%), mean (SD) or median (Q1, Q3). * Pre-bushfire visit median 133 days prior to the 2019/2020 bushfire period. ACQ-5: Juniper Asthma Control Questionaire-5 item; AQLQ(S): Standardized Juniper Asthma Quality of Life Questionnaire; FEV_1_: forced expiratory volume in one second; FVC: forced vital capacity; ICS: Inhaled corticosteroids; LABA: long-acting β agonist; LAMA: long-acting muscarinic antagonist; OCS: oral corticosteroid.

**Table 2:** Multivariable models for the association between any self-reported symptoms during the bushfire period and bushfire smoke event days/bushfire-related PM_2.5_ concentrations (1 October 2019 to 29 February 2020) (*n =* 165).

Variables	Symptoms during the Bushfire Period			
	Crude RR (95%CI)	*p*-Value	Adjusted RR (95%CI)	*p*-Value
Fire day > 41 days	1.02 (0.89–1.16)	0.78	0.98 (0.87–1.11)	0.82
Consecutive fire days (>10 days)	1.03 (0.90–1.17)	0.70	1.0 (0.89–1.12)	0.97
Mean PM_2.5_ (>16 µg/m^3^)	0.98 (0.86–1.11)	0.70	0.95 (0.85–1.07)	0.43
Peak PM_2.5_ (>115 µg/m^3^)	0.98 (0.86–1.12)	0.80	1.01 (0.90–1.14)	0.85

Adjusted for action taken during the bushfire period (stayed indoors/avoided going outdoors, kept windows and doors shut when inside, used a facemask, used an indoor air cleaner/purifier in your home, avoided exercising outdoors and relocated to other areas). PM: Particulate Matter.

**Table 3:** Bushfire smoke exposure and asthma attacks during the 2019/2020 bushfire period compared to the 2018/2019 bushfire period.

Variables	During 2019/2020 Bushfire Period (1 October 2019 to 29 February 2020)	During the 2018/2019 Bushfire Period (1 October 2018 to 1 March 2019)	*p*-Value
**Exposure data**			
Bushfire day †, median (Q1, Q3), *n =* 165	42 (5,43)	2 (0,2)	**<0.001**
Maximum consecutive bushfire days †, median (Q1, Q3), *n =* 165	11 (0,11)	1 (0,1)	**<0.001**
Mean PM_2.5_ (µg/m^3^) †, median (Q1, Q3), *n =* 165	16.4 (11.3, 16.7)	7.7 (7.0, 8.3)	**<0.001**
Peak PM_2.5_ (µg/m^3^) †, median (Q1, Q3), *n =* 165	115.0 (101.3, 191.7)	18.3 (17.4, 22.0)	**<0.001**
**Exposure data categories**			
Bushfire days > 41 §, *n* (%), *n =* 165	90 (54.5)	0 (0.0)	**<0.001**
Maximum consecutive bushfire days >10 §, *n* (%), *n =* 165	85 (51.5)	0 (0.0)	**<0.001**
Mean PM_2.5_ > 16 µg/m^3^ §, *n* (%), *n =* 165	100 (60.6)	0 (0.0)	**<0.001**
Peak PM_2.5_ > 115 µg/m^3^ §, *n* (%), *n =* 165	82 (49.7)	0 (0.0)	**<0.001**
**Experienced an attack of asthma that resulted in:**			
OCS started or increased for at least 3 days *n* (%), *n =* 92	37 (40.2)	28 (30.4)	0.11
Unscheduled Dr visits ‡, *n* (%) *n =* 92	23 (25.0)	9 (9.8)	**0.008**
OCS started or increased, courses † median (Q1, Q3) *n =* 88	0 (0, 2)	0 (0, 1)	0.13
Unscheduled Dr visits †, median (Q1, Q3), *n =* 91	0 (0, 1)	0 (0, 0)	**0.016**

‡ McNemar test; † Wilcoxon signed-rank test; § binomial test. OCS: oral corticosteroid; PM: particulate matter.

**Table 4:** Demographic and clinical characteristics in asthma participants with and without persistent symptoms.

Variables	Total	Persistent Symptoms following Bushfire Period *n* (%)		Crude RR (95% CI)	*p*-Value
		Yes (156)	No (84)		
**Age (years) during the bushfire** **†**	240	63 (50.5, 71.5)	64 (56.0, 70.0)	1.00 (0.99–1.01)	0.45
**Sex**	240				
Male	95	52 (33.3)	43 (51.2)		
Female	145	104 (66.7)	41 (48.8)	1.31 (1.06–1.62)	**0.012**
**Smoking status**	236				
Never smoker	148	89 (57.8)	59 (72.0)		
Smoker (Ex and current)	88	65 (42.2)	23 (28.0)	1.23 (1.02–1.47)	**0.026**
Missing	4	2	2		
**Asthma severity**	240				
Severe asthma	222	145 (93.0)	77 (91.7)	1.07 (0.73–1.56)	0.73
Non-severe asthma	18	11 (7.0)	7 (8.3)		
**Using maintenance OCS**	232				
Yes	49	34 (22.5)	15 (18.5)	1.08 (0.87–1.35)	0.46
No	183	117 (77.5)	66 (81.5)		
Missing	8	5	3		
**Monoclonal antibody use**	234				
Yes	156	97 (63.8)	59 (72.0)	0.88 (0.73–1.06)	0.19
No	78	55 (36.2)	23 (28.0)		
Missing	6	4	2		
**Macrolide use**	231				
Yes	27	21 (14.0)	6 (7.4)	1.23 (0.98–1.54)	0.07
No	204	129 (86.0)	75 (92.6)		
Missing		6	3		
**ACQ-5 score at pre-bushfire visit**	231				
Uncontrolled asthma (ACQ ≥ 1.5)	103	78 (52.7)	25 (30.1)	1.38 (1.14–1.68)	**0.001**
Controlled asthma (ACQ < 1.5)	128	70 (47.3)	58 (69.9)		
Missing	9	8	1		
**Exacerbation at pre-bushfire visit**					
Yes	87	60 (45.1)	27 (36.0)	1.14 (0.93–1.40)	0.19
No	121	73 (54.9)	48 (64.0)		
Missing	32	23	9		
**Lung function**					
FEV_1_ % predicted preB2 < 80	125	83 (72.8)	42 (71.2)	1.03 (0.80–1.31)	0.82
FEV_1_ % predicted preB2 ≥ 80	48	31 (27.2)	17 (28.8)		
Missing	67	42	25		
FVC % predicted preB2 < 100	143	98 (86.7)	45 (76.3)	1.32 (0.91–1.92)	0.14
FVC % predicted preB2 ≥ 100	29	15 (13.3)	14 (23.7)		
Missing	68	43	25		
**Quality of life, AQLQ(S) pre-bushfire visit** **†**	198	5.1 (4.1, 6.1)	6.1 (5.1, 6.8)	0.83 (0.77–0.90)	**<0.001**
Missing	42	29	13		
Asthma triggers total *	225	5.8 (2.1)	5.0 (2.2)	1.06 (1.01–1.10)	**0.010**
Missing	15	8	7		

† Continuous variable; median(Q1, Q3); * mean (sd). ACQ-5: Juniper Asthma Control Questionnaire-5-item; AQLQ(S): standardized Juniper Asthma Quality of Life Questionnaire; B2: bronchodilator; FEV_1_: forced expiratory volume in one second; FVC: forced vital capacity; OCS: oral corticosteroid.

**Table 5:** Multivariable models for the association between persistent symptoms following the bushfire period and bushfire smoke event days/bushfire-related PM_2.5_ concentrations (1 October 2019 to 29 February 2020), stratified by (**a**). monoclonal antibody use before the bushfire period (*n =* 162) and (**b**). asthma symptom control prior to the bushfire period (*n =* 157).

**Variables**	**Persistent Symptoms**				
(**a**).	**mAb User**		**mAb Non-User**		
	**aRR**	***p*-Value**	**aRR**	***p*-Value**	**P for Interaction**
Fire day > 41 days	1.09 (0.84–1.42)	0.51	0.65 (0.47–0.90)	**0.009**	**0.015**
Consecutive fire day (>10 days)	1.08 (0.83–1.40)	0.59	0.77 (0.53–1.13)	0.18	0.24
Mean PM_2.5_ (>16 µg/m^3^)	0.90 (0.69–1.17)	0.44	0.67 (0.48–0.95)	**0.023**	0.33
Peak PM_2.5_ (>115 µg/m^3^)	0.77 (0.60–0.99)	**0.046**	1.69 (1.26–2.26)	**<0.001**	**<0.001**
**Variables**	**Persistent symptoms**				
(**b**).	**Controlled asthma**		**Uncontrolled asthma**		
	**aRR**	***p*-value**	**aRR**	***p*-value**	**P for Interaction**
Fire day > 41 days	1.25 (0.87–1.79)	0.23	0.84 (0.64–1.10)	0.21	0.07
Consecutive fire day (>10 days)	1.18 (0.83–1.68)	0.36	1.0 (0.79–1.26)	0.99	0.26
Mean PM_2.5_ (>16 µg/m^3^)	1.0 (0.71–1.40)	0.99	0.86 (0.67–1.11)	0.25	0.44
Peak PM_2.5_ (>115 µg/m^3^)	0.66 (0.45–0.96)	**0.031**	1.15 (0.91–1.46)	0.24	**0.009**

Adjusted for action taken during the bushfire period (stayed indoors/avoided going outdoors, kept windows and doors shut when inside, used a facemask, used an indoor air cleaner/purifier in your home, avoided exercising outdoors and relocated to another area). mAb: monoclonal antibody; PM: particulate matter; aRR: adjusted relative risk. Controlled asthma is Asthma Control Questionnaire-5 < 1.5 and uncontrolled asthma is Asthma Control Questionnaire-5 ≥ 1.5.

## Data Availability

Data sharing not applicable as this was not approved by the institutional ethics committee.

## Supplementary Materials

The following supporting information can be downloaded at: https://www.mdpi.com/article/10.3390/ijerph19127419/s1, Figure S1: Location of participants and fixed site government air quality monitoring stations in the Sydney Greater Metropolitan Region (New South Wales) study regions; Figure S2: Location of participants and fixed site government air quality monitoring stations in the Melbourne (Victoria) study region; Figure S3: Directed acyclic graph (DAG) showing the assumed relationships among exposure to fine particulate matter (fire day/maximum consecutive fire days/mean PM_2.5_), self-reported persistent symptoms and other related factors; Table S1: Sensitivity analysis for association between persistent symptoms and bushfire smoke event days/bushfire-related PM_2.5_ concentrations 1 October 2019 to 29 February 2020; Table S2: Multivariable models for the association between persistent symptoms and bushfire smoke event days/bushfire-related PM_2.5_ concentrations stratified by sex 1 October 2019 to 29 February 2020; Table S3: Persistent symptoms and smoke exposure mitigation actions taken during the 2019/2020 bushfire season.
