# Supplementary material for: ‘Breathing Fire’: Impact of Prolonged Bushfire Smoke Exposure in People with Severe Asthma
Source: Int J Environ Res Public Health. 2022 Jun 16;19(12):7419. doi: 10.3390/ijerph19127419 (PMC9224478; doi:10.3390/ijerph19127419)
Supplement: Supplementary file 1 [file ijerph-19-07419-s001.zip › ijerph-1734948-supplementary.pdf]

# SUPPLEMENTARY MATERIALS *for:*

**TITLE: 'Breathing Fire': Impact of Prolonged Bushfire Smoke Exposure in People with Severe Asthma**

**Authors:** Tesfalidet Beyene*[1], Erin S. Harvey*[1,2], Joseph Van Buskirk[3], Vanessa M. McDonald [2,4], Megan E. Jensen[1], Jay C. Horvat[5], Geoffrey G. Morgan[3], Graeme R. Zosky[6], Edward Jegasothy[3], Ivan Hanigan[3], Vanessa E. Murphy[1], Elizabeth G. Holliday[1], Anne E.Vertigan[1,7], Matthew Peters[8], Claude S. Farah[9], Christine R. Jenkins[8,9], Constance H. Katelaris [10], John Harrington[2], David Langton [11,12], Philip Bardin[13], Gregory P. Katsoulotos[14,15,16], John W. Upham[17,18], Jimmy Chien[19,20], Jeffrey J. Bowden[21], Janet Rimmer [16,22], Rose Bell [23] *and* Peter G. Gibson[1,2]

*These authors contributed equally to this work.

**Authors' affiliation(s):**

*1. School of Medicine and Public Health, The University of Newcastle, Callaghan, NSW Australia*

*2. Department of Respiratory and Sleep Medicine, John Hunter Hospital, Newcastle, NSW Australia*

*3. Sydney School of Public Health, and University Centre for Rural Health, Faculty of Medicine and Health, University of Sydney, NSW, Australia.*

*4. School of Nursing and Midwifery, The University of Newcastle, Callaghan, NSW Australia.*

*5. School of Biomedical Sciences and Pharmacy, The University of Newcastle, Callaghan, NSW Australia.*

*6. Tasmanian School of Medicine, Menzies Institute for Medical Research, University of Tasmania, Hobart, Australia*

*7. Department of Speech Pathology, John Hunter Hospital, Newcastle, NSW Australia*

*8. Department of Thoracic Medicine, Concord Hospital, Concord, Australia.*

*9.Concord Clinical School University of Sydney, Concord, Australia.*

*10. School of Medicine, Western Sydney University, and Campbelltown Hospital, Campbelltown, Australia.*

*11.Faculty of Medicine, Nursing and Health Sciences, Monash University, Clayton, Australia.*

*12. Department of Thoracic Medicine, Frankston Hospital, Frankston, Australia.*

*13. Lung and Sleep Medicine, Monash University and Medical Centre, Clayton, Australia.*

*14. St George Specialist Centre, Kogarah, Australia.*

*15. St George and Sutherland Clinical School, University of New South Wales, Sydney, Australia.*

*16.Woolcock Institute of Medical Research, Glebe, Australia.*

*17. Department of Respiratory Medicine, Princess Alexandra Hospital, Woolloongabba, Australia.*

*18. The University of Queensland Diamantina Institute, Woolloongabba, Australia*

*19. Department of Respiratory and Sleep Medicine, Westmead Hospital, Westmead, Australia.*

*20. School of Medicine, The University of Sydney, Sydney, Australia.*

*21. Respiratory and Sleep Services, Flinders Medical Centre and Flinders University, Bedford Park, Australia.*

*22. St Vincent's Clinic, Darlinghurst, Australia*

*23. Asthma Australia, Melbourne, VIC Australia*

## ADDITIONAL METHODS

### Exposure measures

*Particulate matter ($PM_{2.5}$) estimates:* The Sydney study region consisted of the Greater Metropolitan Region (GMR) of Sydney. The Melbourne study region consisted of a 100km radius of the Melbourne Central Business District, due to fewer Victorian $PM_{2.5}$

monitoring stations being available. These regions were similar in area. The region average $PM_{2.5}$ levels were obtained from 20 to 25 monitoring stations in the Sydney GMR and 4 stations in the Melbourne study region (Figures S1, S2). The $PM_{2.5}$ estimates for each region differed in the number of monitors that contribute to the interpolation, and the spatial distribution of those monitors, affecting the distribution of the estimates in each region, however not substantially affecting the individual participant $PM_{2.5}$ estimates.

Overall, 108 participants (56%) had at least one listed address within the Sydney GMR study region, with an additional 57 participants having at least one listed address in the Melbourne study region, allowing estimation of $PM_{2.5}$ for a total of 165/240 participants (69%). Most of the participants in the Melbourne study region were located close to the Melbourne Central Business District, whilst the participants in the Sydney GMR study region were located across the study region.

*Bushfire day identification:* Due to the high stringent requirements for bushfire identification, a lag tolerance of two days for bushfire events was permitted. That is, if two identified bushfire days were separated by one or two non-bushfire event days that exceeded the 95$^{th}$ percentile for $PM_{2.5}$, these additional days were also coded as bushfire events.

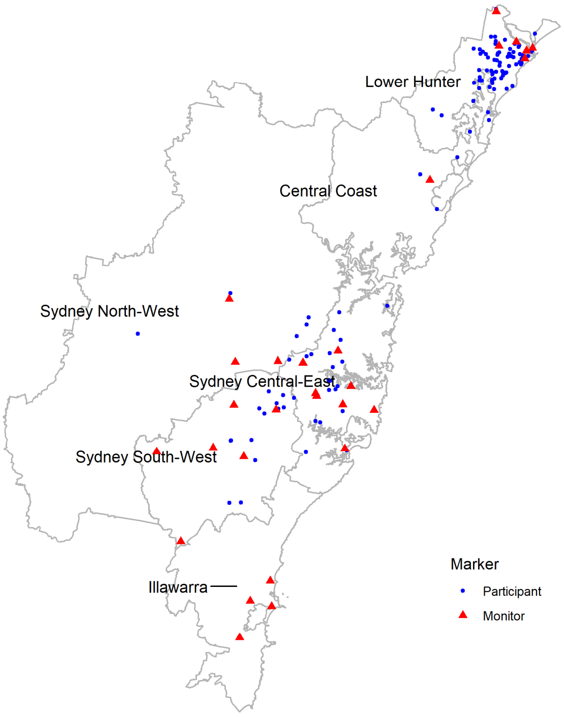


**Figure S1: Location of participants and fixed site government air quality monitoring stations in the Sydney Greater Metropolitan Region (New South Wales) study regions.** New South Wales Department of Planning, Industry and Environment and Environmental Protection Agency, Victoria.

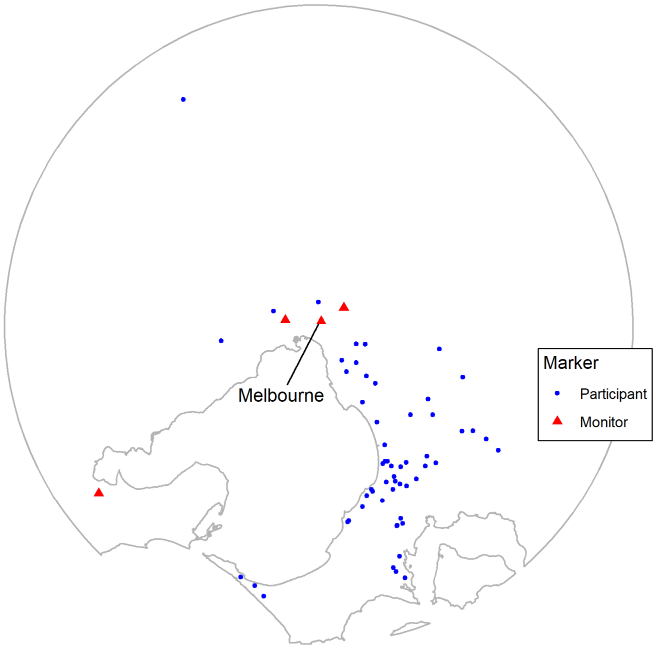


**Figure S2: Location of participants and fixed site government air quality monitoring stations in the Melbourne (Victoria) study region.** Environment Protection Authority, Victoria**.**

## Statistical analysis

Data for continuous variables were summarised using mean with standard deviation (SD) or median with interquartile range (Q1,Q3). Data for categorical variables were summarised using frequency with percent. Bivariate analyses were performed to determine the association between participant characteristics and persistent symptoms. Wilcoxon signed-rank sum test were used to test the within-person difference in median number of asthma attacks between the 2019/2020 bushfire period compared to the pre-bushfire visit and 2018/2019 bushfire period. McNemar's test was performed to assess the difference between paired categorical variables.

*Directed Acyclic Graph (DAG):* Using the DAG (Figure S3), we identified the minimum adjustment set of potential confounding variables required to estimate the effect of bushfire smoke exposure on self-reported asthma symptoms. Ancestor of exposure is green, ancestor of both exposure and outcomes are red, green arrow indicates open casual path, blue circle indicates outcome variable and open circle refers to adjusted variables.

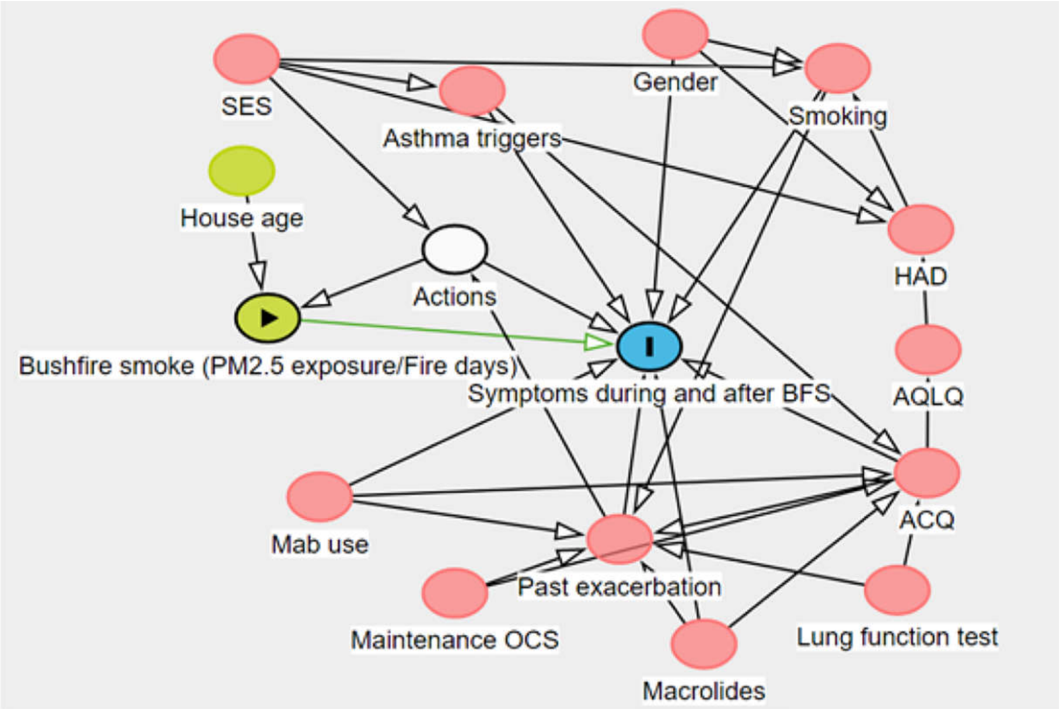


**Figure S3: Directed acyclic graph (DAG) showing the assumed relationships among exposure to fine particulate matter (fire day/maximum consecutive fire days/ mean $PM_{2.5}$), self-reported persistent symptoms and other related factors.**

*Secondary analyses*: We conducted stratified analyses within categories of putative effect modifiers of monoclonal antibody use at the pre-bushfire visit, asthma symptom

control at the pre-bushfire visit. Effects estimated within strata were reported as Relative Risks (RR) with 95% Confidence Interval (CI), together with the type III p-value for interaction between the exposure and relevant effect modifier, estimated using a separate model.

## ADDITIONAL RESULTS

### Secondary analyses:

We conducted stratified analyses within categories of putative effect modifiers of gender, monoclonal antibody uses at the pre-bushfire visit, asthma symptom control (ACQ-5) and exacerbations at the pre-bushfire visit. The sensitivity analysis (Table S1) revealed no marked difference in exposure effect estimates when other covariates were fitted to the multivariable model, suggesting the minimum adjustment set identified by the DAG was sufficient for confounding adjustment.

*Sensitivity analysis*

**Table S1:** ***Sensitivity analysis*** **for association between** ***persistent symptoms*** **and bushfire smoke event days/ bushfire related $PM_{2.5}$ concentrations 1 October 2019 to 29 February 2020. (n=118)**

| Variables | Persistent symptoms | | | |
|---|---|---|---|---|
| | Crude RR (95%CI) | p-value | Adjusted RR (95%CI) | p-value |
| Fire day >41 days | 0.99 (0.81 - 1.22) | 0.95 | 1.05 (0.81 - 1.35) | 0.72 |
| Consecutive fire day (>10days) | 1.08 (0.88 – 1.33) | 0.45 | 1.09 (0.84 - 1.42) | 0.51 |
| Mean $PM_{2.5}$ (>16) | 0.89 (0.73 - 1.09) | 0.28 | 0.92 (0.70 - 1.20) | 0.53 |
| Peak $PM_{2.5}$ (>115) | 0.91 (0.74 - 1.12) | 0.37 | 0.95 (0.74 - 1.21) | 0.67 |

*Adjusted for action taken during the bushfire, gender, smoking status, Mab use, macrolide use, ACQ5 at pre-bushfire visit, exacerbation at pre-bushfire visit, Juniper AQLQ at pre-bushfire visit, asthma. PM: Particulate Matter

*Stratified analysis:*

*Symptoms by sex*

**Table S2: Multivariable models for the association between *persistent symptoms* and bushfire smoke event days/bushfire related $PM_{2.5}$ concentrations stratified by *sex* 1 October 2019 to 29 February 2020. (n=165)**

| Variables | Persistent symptoms | | | | |
|---|---|---|---|---|---|
| | Male | | Female | | |
| | ARR | p-value | ARR | p-value | **P for Interaction** |
| Fire day >41 days | 0.98 (0.64 - 1.51) | 0.93 | 0.97 (0.77 - 1.22) | 0.80 | 0.93 |
| Consecutive fire day (>10days) | 1.16 (0.75 - 1.78) | 0.51 | 1.01 (0.80 - 1.27) | 0.93 | 0.52 |
| Mean $PM_{2.5}$ (>16) | 0.87 (0.57 - 1.32) | 0.51 | 0.86 (0.69 - 1.08) | 0.20 | 0.87 |
| Peak $PM_{2.5}$ (>115) | 1.11 (0.73 - 1.68) | 0.63 | 0.86 (0.68 - 1.09) | 0.20 | 0.35 |

*Adjusted for action taken during the bushfire period (stayed indoors/avoided going outdoors, kept windows and doors shut when inside, used a facemask, used an indoor air cleaner/purifier in your home, avoided exercising outdoors and relocated to another areas). PM: Particulate Matter

## Symptoms by actions taken

**Table S3: Persistent symptoms and smoke exposure mitigation actions taken during the 2019/2020 bushfire season**

| Actions taken | Total | Persistent symptoms Yes (156) | No (84) | p-value |
|---|---|---|---|---|
| Stayed indoors/avoided going outdoors | **240** | | | |
| Yes | 212 | 143 (91.7) | 69 (82.1) | **0.028** |
| No | 28 | 13 (8.3) | 15 (17.9) | |
| Kept windows and doors shut when inside | | | | |
| Yes | 223 | 147 (94.2) | 76 (90.5) | 0.28 |
| No | 17 | 9 (5.8) | 8 (9.5) | |
| Used a face mask | | | | |
| Yes | 49 | 32 (20.5) | 17 (20.2) | 0.96 |
| No | 191 | 124 (79.5) | 67 (79.8) | |
| Used an air conditioner at home | | | | |
| Yes | 173 | 116 (74.4) | 57 (67.9) | 0.28 |
| No | 67 | 40 (25.6) | 27 (32.1) | |
| Used an indoor air cleaner/purifier at home | | | | |
| Yes | 27 | 19 (12.2) | 8 (9.5) | 0.53 |

| | | | | |
|---|---|---|---|---|
| No | 213 | 137 (87.8) | 76 (90.5) | |
| Avoided exercising outdoors | | | | |
| Yes | 199 | 138 (88.5) | 61 (72.6) | **<0.001** |
| No | 41 | 18 (11.5) | 23 (27.4) | |
| Relocated to another area | | | | |
| Yes | 11 | 9 (5.8) | 2 (2.4) | 0.33 |
| No | 229 | 147 (94.2) | 82 (97.6) | |

Data presented as N(%)
